# Identification of a Prognostic Pyroptostic-Related Model for Head and Neck Squamous Cell Carcinoma Based on LASSO-Cox Regression Analysis

**DOI:** 10.1155/2022/1434565

**Published:** 2022-11-22

**Authors:** Donglei Wu, Wanting Fan, Zheng Zhang, Jianming Tang, Chenping Zhang, Weixuan Chen, Nianhong Qin, Yuyan Zheng

**Affiliations:** ^1^Department of Stomatology, Shenzhen People's Hospital, Shenzhen, China; ^2^Department of Anesthesia, The First Affiliate Hospital of Guangzhou Medical University, Guangzhou, China

## Abstract

Pyroptosis is associated with the biological behavior of the tumor and with tumor immunity. We investigated the effect of pyroptosis on the tumor microenvironment and tumor immunity in head and neck squamous cell carcinoma (HNSCC). RNA sequencing data and clinical information of HNSCC were downloaded from TCGA. Differentially expressed pyroptosis-related genes in HNSCC were identified between HNSCC and normal tissue. Pyroptosis-related classification of HNSCC was conducted based on consensus clustering analysis. LASSO-Cox regression analysis was used to construct a prognostic risk model-based pyroptosis-related gene. Evaluation of the immune microenvironment was conducted in prognostic risk signature based on pyroptosis-related genes. Total 22 differentially expressed pyroptosis-related genes were identified in HNSCC. Six prognostic-related genes were included to construct a LASSO regression model with a prognostic risk score = (0.133 ∗ GSDME (DFNA5) + 0.084 ∗ NOD1 + 0.039 ∗ IL6 + 0.003 ∗ IL1B + 0.084 ∗ CASP3 + 0.028 ∗ NLRP2). Higher fraction of resting memory CD4+ T cells and macrophages M1 was infiltrated in the high-risk group compared with the low-risk group in HNSCC. Furthermore, the PI3K-Akt signaling pathway and the IL-17 signaling pathways were identified to be involved in the development of high-risk HNSCC. Our study constructed a prognostic risk signature based on pyroptosis-related genes, which emphasizes the critical importance of pyroptosis in HNSCC and provided a novel perspective of HNSCC therapy.

## 1. Introduction

Head and neck squamous cell carcinoma (HNSCC) ranks as the seventh most common cancer worldwide, which leads to 450,000 deaths in 2018 [1, 2]. Tobacco use, alcohol consuming, and HPV infection are the main risk factors, especially HPV-positive associated HNSCC, which is increasing among young people in the western world [1]. Multiple therapeutic interventions, including surgery, radiation therapy, chemotherapy, and immunotherapy, are recommended for HNSCC according to the anatomical sites and the degree of severity of HNSCC [1]. Despite tremendous efforts to therapy intervention for HNSCC, low therapeutic response to HNSCC remains a global challenge [3]. The prognosis in HNSCC varies dramatically depending on risk factors, perineural invasion and extensive of invasion [4]. In addition, recurrent and metastatic HNSCC still have a poor prognosis [5]. Therefore, understanding the tumor biology of HNSCC will help us predict the prognosis and identify novel therapeutic targets for HNSCC. Recently, pyroptosis in cancer cell death induced by targeted therapy ad chemotherapy has gained more and more attention in cancer management [6, 7]. Pyroptosis, a kind of regulated cell death pathway [8], is characterized by a distinct inflammatory outcome, accompanied by the release of many inflammatory cytokines release. It presents with cellular swelling and rapid rupture. The Gasdermin (GSDM) superfamily, including GSDMA, GSDMB, GSDMC, GSDMD, and GSDME (DFNA5), are activated and cleaved during pyroptosis, which is regulated by inflammatory caspases [8]. Several studies have demonstrated that pyroptosis increases cancer immune therapy [9–12]. However, the role of pyroptosis in HNSCC remains unclarified. In this study, our objective was to identify the signature to predict the prognosis of HNSCC and help provide a novel therapy strategy for HNSCC.

## 2. Materials and Methods

### 2.1. Data Preprocess

RNA sequencing data and clinical information of HNSCC was downloaded from TCGA (https://tcga-data.nci.nih.gov) database. Total 542 HNSCC and 44 normal human samples were included in this study. The gene expression profiles in HNSCC were normalized to fragment per kilobase million (FRKM).

### 2.2. Identification of Differentially Expressed Pyroptosis-Related Genes in HNSCCs

Thirty-three pyroptosis-related genes were retrieved from the published research works [9–12], which included effector substrate proteins-six GSDM superfamily members (GSDMA, GSDMB, GSDMC, GSDMD, GSDME (DFNA5), and DFNB59), seven caspase family members (CASP1, CASP3, CASP4, CASP5, CASP6, CASP8, and CASP9), pattern recognition receptors, including eight nucleotide-binding oligomerization domain-like receptors (NOD1, NOD2, NLRP1, NLRP2, NLRP3, NLRP6, NLRP7, and NLRC4), absent in melanoma 2(AIM2), released inflammatory cytokine (IL-1B, IL-6, IL-18, and TNF) and other regulatory genes (GPX4, PLCG1, PYCARD, PRKACA, ELANE, TIRAP, and SCAF11).

Differentially expressed pyroptosis-related genes (DEPG) between HNSCC and normal tissues were identified by “limma” package. The heatmap and bar plots were constructed to depict the expression of DEPG. Furthermore, the correlation among 33 genes in HNSCC was conducted based on Pearson's correlation analysis. A *P* value less than 0.05 was considered significance.

### 2.3. Pyroptosis-Related Classification of HNSCC Based on Consensus Clustering Analysis

To further identify the pyroptosis-related subtype of HNSCC, consensus clustering analysis was performed to justify the classification of HNSCC based on the resampling method. A consensus matrix was clustered to determine the optimal *k* value. In addition, the clinicopathological characteristics (including age, sex, grade, stage of TNM, metastasis, and alcohol use) of HNSCC were included in the heatmap. To further evaluate the effect of pyroptosis-related classification on survival time in HNSCC, survival analysis was performed between two pyroptosis-related clusters in HNSCC. The survival rate was estimated on the basis of the Kaplan–Meier method. Comparison of the survival rate between two clusters was implemented with the log-rank test. A *P* value less than 0.05 was considered significance.

### 2.4. Construction of a Risk Signature of HNSCC in TCGA

To further identify the prognostic genes of pyroptosis, univariate Cox regression analysis was applied to identify the prognostic genes of pyroptosis-related genes. Subsequently, the prognostic pyroptosis-related genes were enrolled in the LASSO-Cox regression analysis to avoid overfitting the model. The contraction penalty term (*λ*) was introduced into the model to optimize the regression model. Five-fold cross-validation was used to determine the optimal *λ* by choosing the logarithm of the minimum mean squared error of the lambda. LASSO-Cox-regularized regression analysis was performed using “glmnet” package in R. The ROC curve was conducted to evaluate the efficacy of the prognostic risk model of HNSCC with pyroptosis-related genes.

### 2.5. Evaluation of the Immune Microenvironment in a Prognostic Risk Signature Based on Pyroptosis-Related Genes

To further evaluate the association between the immune microenvironment and prognostic risk model based on pyroptosis-related genes in HNSCC, cell-type identification by estimating relative subsets of RNA transcripts (CIBERSORT) and the ESTIMATE method were applied to analyze the infiltration of immune and stroma cells [13]. CIBERSORT (https://cibersort.stanford.edu/) calculates 22 immune cells based on the deconvolution method. ESTIMATE (https://bioinformatics.mdanderson.org/estimate/) was applied to calculate the scores of stromal and immune cells.

### 2.6. Functional Enrichment of Differentially Expressed Genes between High Risk and Low Risk Group at HSNCC

Differentially expressed genes (DEG) between the high-risk and low-risk groups in HSNCC were calculated using “limma” package with absolute log2(|fold change|) >1 and *P* value <0.05. To further understand the potential mechanism in DEG between the high-risk and low-risk groups in HNSCC, functional enrichment of Gene Ontology (GO) (https://geneontology.org/) and Kyoto Encyclopedia of Genes and Genomes (KEGG) (https://www.kegg.jp/) was applied to identify the biological function of hub genes in the significant module.

## 3. Results

### 3.1. Identification of DEPG between HNSCC and Normal Tissues

**Figure 1:**
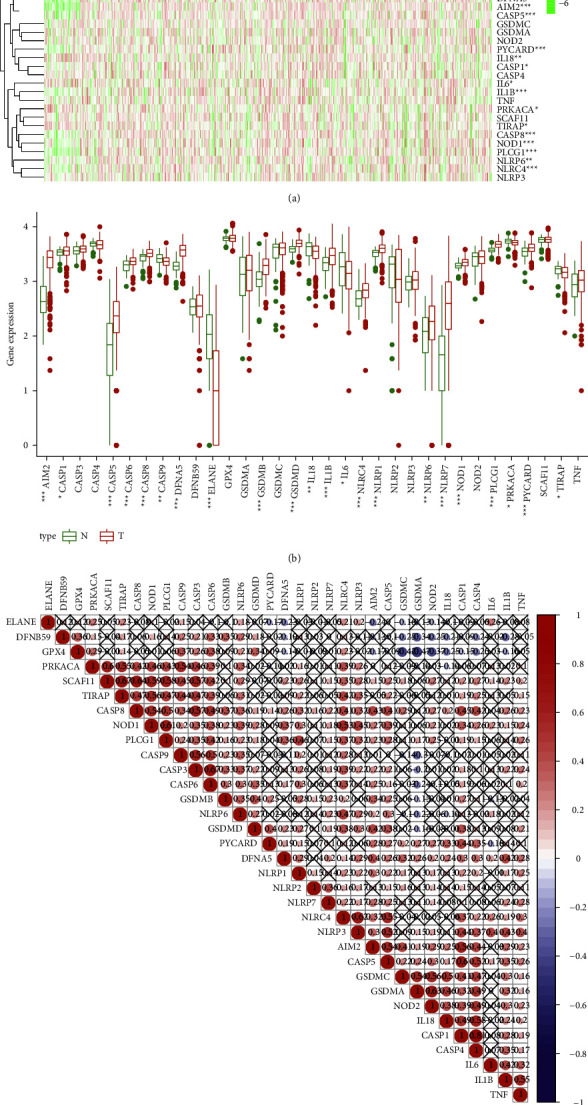
Differentially expressed pyroptosis-related genes between HNSCC and normal tissue. (a) The heatmap of 33 pyroptosis-related gene expression between HNSCC and normal tissue. (b) Bar plot of 33 pyroptosis-related gene expressions between HNSCC and normal tissue. *N* means normal tissues; *T* means HNSCC tissues; ^∗^, compared with normal tissue, *P* < 0.05; ^∗∗^*P* < 0.01; ^∗∗∗^*P* < 0.001. (c) The heatmap of correlation among 33 pyroptosis-related genes in HNSCC, the red dots mean positive correlation and blue dots mean positive correlation (*n* = 582).

As shown in Figure 1(a), 22 DEPGs (including AIM2, CASP1, CASP5, CASP6, CASP8, CASP9, GSDME(DFNA5), ELANE, GSDMB, GSDMD, IL-1B, IL-6, IL-18, NLRC4, NLRP1, NLRP6, NLRP7, NOD1, PLCG1, PRKACA, PYCARD, and TIRAP) were identified between the HNSCC tissue and normal tissue. Higher expression of AIM2, CASP1, CASP5, CASP6, CASP8, GSDME (DFNA5), GSDMB, GSDMD, IL-18, IL6, NLRC4, NLRP6, NLRP7, NOD1, PLCG1, and PYCARD in HSNCC tissues compared with the normal tissues, whereas a few pyroptosis-related genes (CASP9, ELANE, IL-1B, NLRP1, TIRAP, and PRKACA) were downregulated in HNSCC tissues (*P* < 0.05) (Figure 1(b)). Pearson correlation analysis indicated a highly positive correlation between CASP4 and CASP1 (*r* = 0.81, *P* < 0.05). Moderate positive correlations between PRKACA and SCAF11 (*r* = 0.6, *P* < 0.05), SCAF11 and TIRAP (*r* = 0.67, *P* < 0.05), SCAF11 and CASP8 (*r* = 0.64, *P* < 0.05), and CASP3 and CASP6 (*r* = 0.67, *P* < 0.05) are manifested in Figure 1(c).

### 3.2. Identification of Pyroptosis-Related Subtype of HNSCC Based on Consensus Cluster Analysis

**Figure 2:**
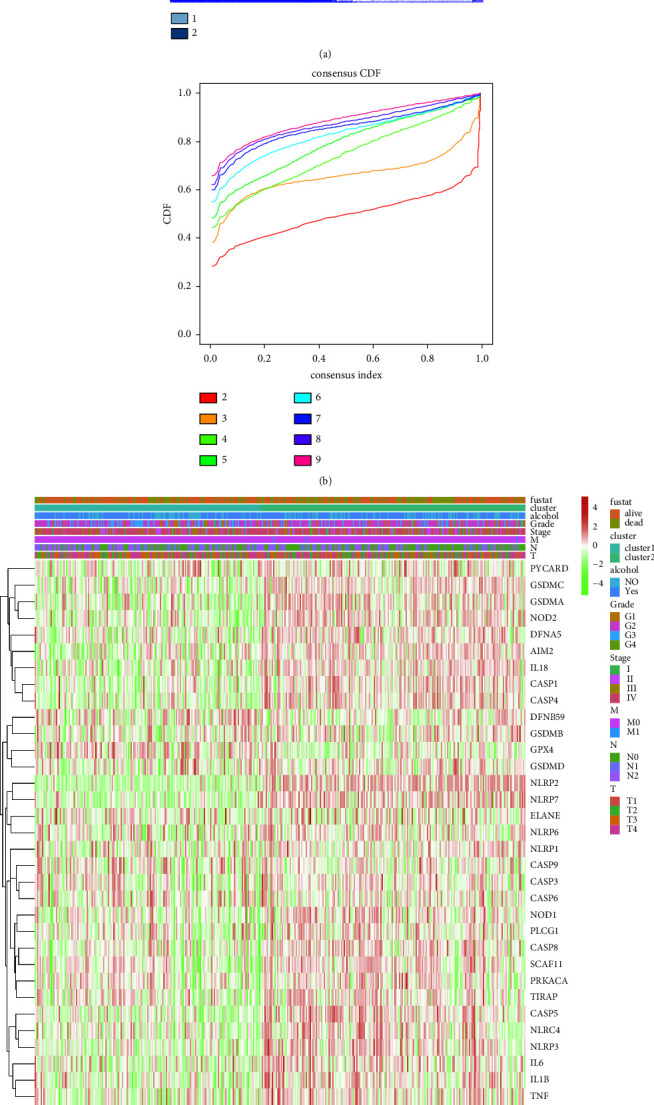
Identification of pyroptosis-related subtype of HNSCC based on consensus cluster analysis. (a) Consensus matrix plot of HNSCC. The classification of HNSCC with optimal *k* = 2. (b) Empirical cumulative distribution function (CDF) plots. The CDF plot finds that the *k* = 2 at which the distribution reaches an approximate maximum. (c) The heatmap of expression of 33 pyroptosis-related genes between the two clusters and among clinicopathologic features. (d) Survival analysis between the 2 pyroptosis-related clusters of HNSCC (*n* = 542).

The pyroptosis-related subtype of HNSCC was clustered into two groups based on consensus clustering analysis (*k* = 2) (Figures 2(a) and 2(b)). A higher level of 33 pyroptosis-related genes was clustered in cluster 2 (Figure 2(c)). In addition, a higher survival rate was detected in cluster 1 of HNSCC compared with cluster 2 (*P* < 0.001) (Figure 2(d)).

### 3.3. Construction of a Prognostic Risk Model with HNSCC Based on Pyroptosis-Related Genes

**Figure 3:**
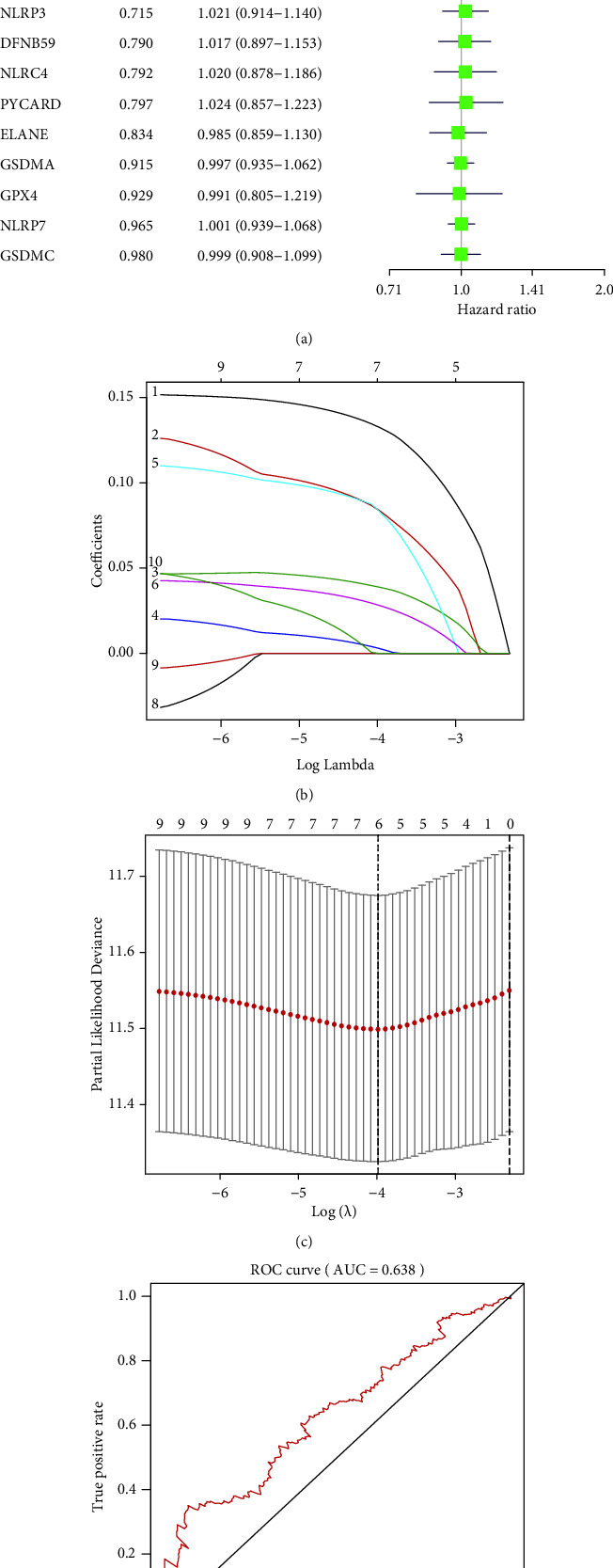
Construction of a prognostic risk model with pyroptosis-related genes. (a) Univariate cox regression coefficients in 33 pyroptosis-related genes in HNSCC. (b) Identification of the penalty parameters for the pyroptosis-related genes. (c) Cross-validation plot for the term of penalty. (d) ROC curves for the prognostic risk model. AUC, area under the curve. (e) Survival curve between the high-risk and low-risk groups in prognostic risk model of HNSCC (*n* = 542).

As shown in Figure 3(a), only 4 pyroptosis-related genes (including GSDME (DFNA5), IL-6, IL-1B, and CASP3) were considered significant prognostic-related genes with univariate cox regression analysis (*P* < 0.05). LASSO-Cox regression analysis indicated that a prognostic risk model was constructed by 6 prognostic-related genes, including GSDME (DFNA5), NOD1, IL-6, IL-1B, NLRP2, and CASP3 (Figure 3(b)). The optimal lambda was identified as 4 by the cross-validation analysis (Figure 3(c)). Hence, the computational formula of prognostic-related risk score in HNSCC was as follows: risk score=(0.133 ∗ GSDME + 0.084 ∗ NOD1 + 0.039 ∗ IL6 + 0.003 ∗ IL1B + 0.084 ∗ CASP3 + 0.028 ∗ NLRP2). A moderate efficiency was verified with an AUC of 0.638 from the ROC curve (Figure 3(d)).

### 3.4. Relationship between Prognostic Risk Model and Clinical Characteristics in HNSCC Based on Six Genes

**Figure 4:**
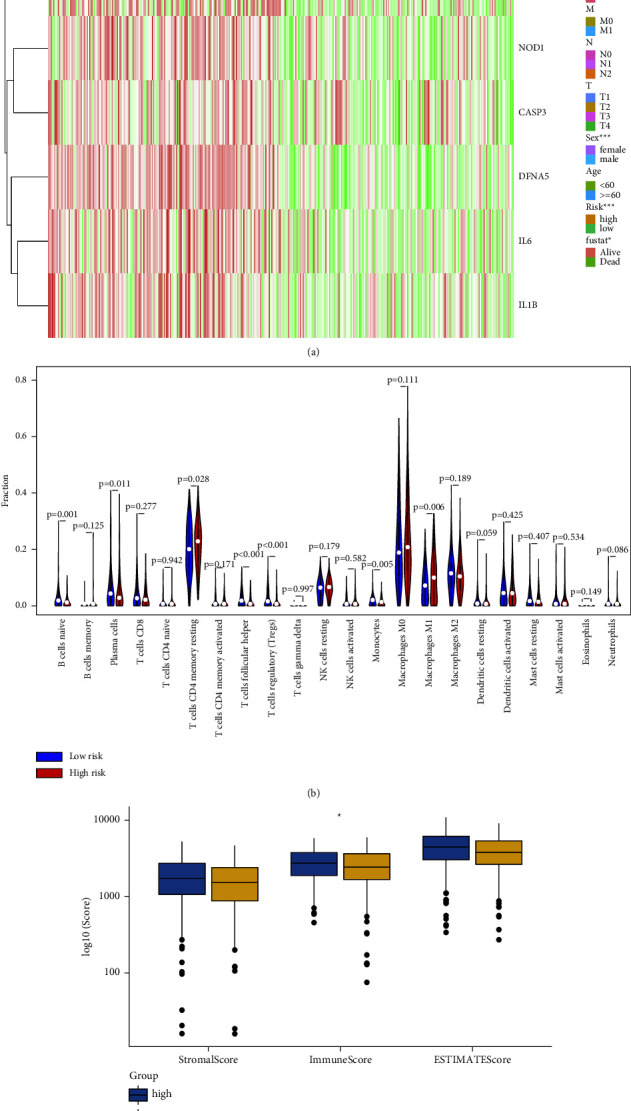
The immune microenvironment of HNSCC between high-and low-risk signature for prognosis. (a) The heatmap of the expression of 6 genes in prognostic risk model. ^∗^*P* value <0.05; ^∗∗∗^*P* value <0.001; red means upregulation; green means downregulation (b). CIBERSORT analysis of 22 immune cell subtypes between the high-risk and low-risk groups in HNSCC. Blue bar for the low-risk group, red bar for the high-risk group. (c). ESTIMATE analysis of the immune score between high- and low-risk signatures in HNSCC. ^∗^*P* value <0.05 (*n* = 542).

The HNSCC patients were divided into 2 groups based on the above risk score (high-risk and low-risk group). The HNSCC patient in the high-risk group tended to have a shorter survival time (*P* < 0.001) (Figure 3(e)). In addition, the expression of 6 pyroptosis-related genes (GSDME, IL6, IL1B, NOD1, CASP3, and NLRP2) in the prognostic risk model was elevated in the high-risk group. Compared with the low-risk group, a high-risk group manifested in higher grade with female predominance (*P* < 0.05). However, no differences in alcohol use were observed between the high-risk group and the low-risk group (*P* > 0.05). (Figure 4(a)).

### 3.5. Effects of Pyroptosis-Related Genes on Immune Microenvironment in HNSCC

Immune cell infiltration varied between high and low prognostic risk signature based on pyroptosis-related genes (*P* < 0.05) (Figure 4(b)). A higher fraction of resting memory CD4+ T cells and macrophages M1 was infiltrated in the high-risk group (*P* < 0.05) (Figure 4(b)). Interestingly, a higher fraction of plasma cells, native B cells, native CD4+ T cells, follicular helpers of T cells, and monocytes were identified in the low-risk group (*P* < 0.05). Furthermore, compared with the low-risk group, a higher immune score was detected in the high-risk group of HNSCC (*P* < 0.05) (Figure 4(c)).

### 3.6. Functional Enrichment of DEGs between the High-Risk and Low-Risk Groups in HSNCC

**Figure 5:**
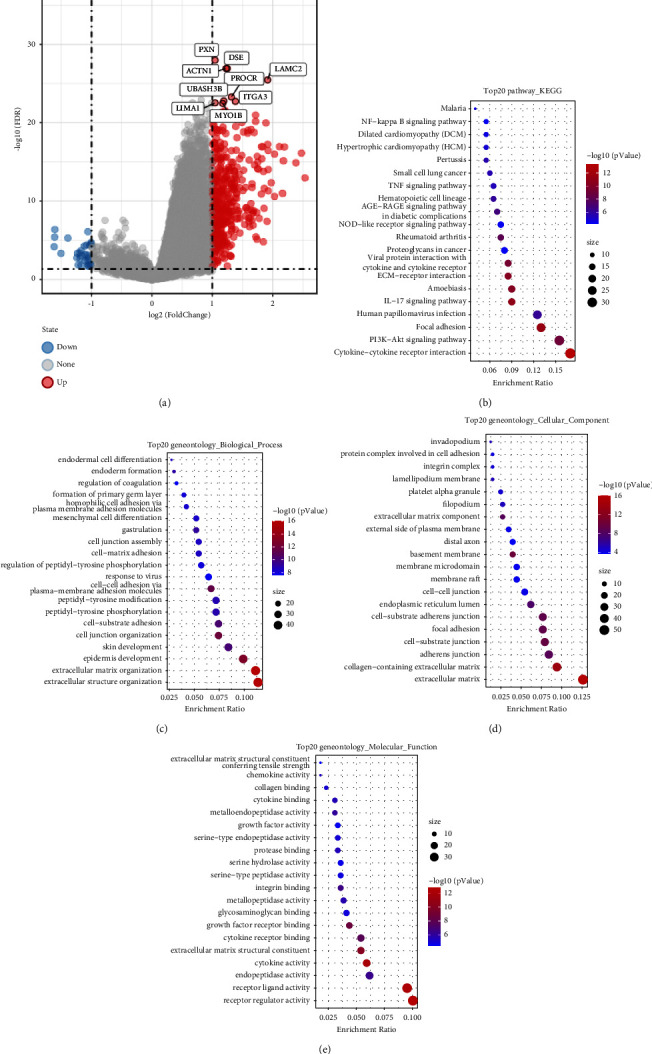
Functional enrichment of differentially expressed genes between the high-risk and low-risk groups in HSNCC. (a) Volcano plot of differentially expressed genes between the high-risk and low-risk group in HSNCC. Blue dot, upregulated genes compared with the low-risk group. Red dot, downregulated genes compared with the low-risk group with |log (FDR)| >1. (b) Dot plot for the top 20 signaling pathways in KEGG analysis; (c) dot plot for the top 20 terms of molecular function in GO analysis; (d) dot plot for the top 20 terms of biological process in GO analysis; (e) dot plot for the top 20 terms of cellular component in GO analysis (*n* = 542).

Higher expression of (GSDME (DFNA5), PXN, ACTN1, DSE, LAMC2, PROCR, UBASH3B, ITGA3, LIMA1, and MYO1B) was identified in the high-risk group compared with the low-risk group (Figure 5(a)). GO and KEGG analyses were performed to evaluate the functional enrichment in DEGs. In KEGG analysis, cytokine-cytokine receptor interaction pathway, phosphatidylinositol 3-kinase (PI3K)-Akt signaling pathway, focal adhesion, and IL-17-signaling pathway were mostly enriched (Figure 5(b)). In the GO analysis, biological processes (including extracellular structure organization and extracellular matrix organization) were mostly enriched (Figure 5(c)). The extracellular matrix, especially for collagen-containing extracellular matrix, was the leading cellular component (Figure 5(d)). In addition, the molecular function among DEGs varies in receptor regulator activity, receptor ligand activity, and endopeptidase activity (Figure 5(e)).

## 4. Discussion

Although pyroptosis is essential for host defense infection and hazardous signals, its role in tumor activity remains ambiguous. Emerging evidence shows that pyroptosis inhibits tumor growth and migration [7]. However, several lines of evidence support that a possible harmful role of pyroptosis in HNSCC [7]. In consistent with our study, high expression of NLR family pyrin domain family members was detected in the HNSCC tissue. Increased NLRP3 is positively correlated with the growth and metastasis of oral squamous cell carcinoma (OSCC) [14, 15]. Knockdown of *Nlrp3* expression inhibits the proliferation, migration, and invasion of OSCC by decreasing the cleavage of Caspase-1 and lL-1*β* release *in vitro* and *in vivo* [14]. In addition, high expression of NLRP3 implied a poor prognosis and minor differentiation in histopathological grading in patients with OSCC. Increased expression of NLRP3 enhances the 5-FU resistance of oral squamous cell carcinoma (OSCC). Knockout of *Nlrp3* and *Caspase1* inhibited the tumor growth in mice with 5-FU [15].

NLRP3 promotes the epithelial-mesenchymal transition (EMT) in colon cancer [16]. Similarly, activation of the NLRP3 inflammasome accelerates tumor proliferation and migration in lung cancer [17]. In addition, the NLRP3 inflammasomes are involved in radiotherapy resistance in glioblastoma [18]. Hence, pyroptosis might play an important role in tumorigenesis in HSNCC.

### 4.1. The Caspase-3/GSDME Signaling Pathway in Head and Neck Cancer

Gasdermin family proteins, as the effectors of pyroptosis, play a critical role in lytic-programmed cell death (pyroptosis) [19, 20]. GSDME-mediated is involved in the regulation of tumor immune microenvironment and antitumor immunity [21]. Consistent with our result, high levels of GSDME have been detected in many cancers, including lung cancer, melanoma, osteosarcoma, digestive cancers, and head and neck cancer [6, 22]. GSDME, cleaved by Caspase 3, releases the pore-forming domain (N-terminal) in the cell membrane to punch holes, which enables the secretion of IL-1B and leading to cell swelling, permeabilization, rupture, and death [22, 23]. In addition, GSDME, the downstream of Caspase 3, is involved in the switch from apoptotic cell death to secondary pyroptosis. Interestingly, GSDME, in turn, enable us to enhance the intrinsic apoptotic pathway in cancer cells by forming pores in the mitochondria with GSDME-N and liberating proapoptotic factors (including Cyt c and HtrA2) [24]. A recent study has indicated that GSDME ablation impaired the tumor-suppressive activity in head and neck cancer [6]. Consistently, LASSO regression model with prognostic risk in our study demonstrated that higher GSDME and CASP3 expression was inclined to a poor prognosis, suggesting a compensatory increase in Caspase-3-mediated cleavage of GSDME in HNSCC.

Not only does GSDME restrains the tumor growth, but augments the function of tumor-infiltrating immune cells [21]. Fewer fractions of tumor-infiltrating lymphocytes and tumor-associated macrophages were detected in the tumor microenvironment when GSDME was knocked out in mice [21]. Similarly, higher number of macrophages and CD4+ T cells was computed from CIBERSORT analysis in our study. In mouse melanoma, the activation of pyroptosis increases the infiltration of tumor-associated T cells and damps dendritic cell infiltrates [25]. In addition, immune microenvironment disorder might induce pyroptosis. Recent studies have demonstrated that serine protease granzymes (Gzmes), transferred by perforin and released by NK cells and cytotoxic T lymphocytes, act to cleave GSDMB and GSDME and lead to pyroptosis [7].

### 4.2. Pattern Recognition Receptors are Involved in the Regulation of Pyroptosis

NOD1 and NLRP2, as the nucleotide-binding oligomerization domain-like receptor (NLPs) members, function as the pattern recognition receptors (PRR) to recognize the pathogen-associated molecular patterns (PAMP) or damage-associated molecular pattern (DAMP) from external stimuli and activate the downstream of caspases through the classical pyroptosis-signaling pathway [26]. Our study demonstrated that NLRP2 was highly expressed in the high-risk group of HNSCC. Similarly, NOD1 and NLRP2 function as risk genes in pyroptosis-related prognostic gene signature related to pyroptosis of lung adenocarcinoma [11]. Recent reports found that NLRP2 elevated the level of profibrotic mediators, but reduced the expression of proinflammatory cytokines and reduced the apoptotic cell rate in proximal tubular epithelial cells [26].

Although the association between pyroptosis and antitumor immunity has been discussed, there is no consensus on the molecular mechanism. PI3K-Akt signaling pathway impacts the tumor growth, migration, survival, and metabolism [27]. Recent research has demonstrated that pyroptosis is induced by ischemia-reperfusion through the PI3K-Akt signaling pathway in neurons [28]. In addition, the activation of the IL-17signaling pathway augments pyroptosis with GSDMD cleave and IL-1 1*β* and IL-18 release in pneumonia-induced sepsis [29]. This evidence supports our results on the role of the IL-17 and PI3K-Akt signaling pathways in high-risk HNSCC.

## 5. Conclusion

Our study constructed a prognostic risk signature based on genes and suggested a role of the PI3K-Akt signaling pathway and the IL-17 signaling pathways in poor survival of HNSCC, which emphasized the critical importance of pyroptosis in HNSCC and provides a novel perspective of the HNSCC therapy.

## Data Availability

The data are available at the TCGA (https://portal.gdc.cancer.gov/).

## References

[1] Chow L. Q. M. (2020). Head and neck cancer. *New England Journal of Medicine*.

[2] Bray F., Ferlay J., Soerjomataram I., Siegel R. L., Torre L. A., Jemal A. (2018). Global cancer statistics 2018: GLOBOCAN estimates of incidence and mortality worldwide for 36 cancers in 185 countries. *CA: A Cancer Journal for Clinicians*.

[3] Bhat A. A., Yousuf P., Wani N. A. (2021). Tumor microenvironment: an evil nexus promoting aggressive head and neck squamous cell carcinoma and avenue for targeted therapy. *Signal Transduction and Targeted Therapy*.

[4] Scanlon C. S., Banerjee R., Inglehart R. C. (2015). Galanin modulates the neural niche to favour perineural invasion in head and neck cancer. *Nature Communications*.

[5] Vermorken J. B., Peyrade F., Krauss J. (2014). Cisplatin, 5-fluorouracil, and cetuximab (PFE) with or without cilengitide in recurrent/metastatic squamous cell carcinoma of the head and neck: results of the randomized phase I/II ADVANTAGE trial (phase II part). *Annals of Oncology*.

[6] Cai J., Yi M., Tan Y. (2021). Natural product triptolide induces GSDME-mediated pyroptosis in head and neck cancer through suppressing mitochondrial hexokinase-ΙΙ. *Journal of Experimental & Clinical Cancer Research*.

[7] Lu X., Guo T., Zhang X. (2021). Pyroptosis in cancer: friend or foe?. *Cancers*.

[8] Koren E., Fuchs Y. (2021). Modes of regulated cell death in cancer. *Cancer Discovery*.

[9] Ye Y., Dai Q., Qi H. (2021). A novel defined pyroptosis-related gene signature for predicting the prognosis of ovarian cancer. *Cell Death & Disease*.

[10] Shao W., Yang Z., Fu Y. (2021). The pyroptosis-related signature predicts prognosis and indicates immune microenvironment infiltration in gastric cancer. *Frontiers in Cell and Developmental Biology*.

[11] Lin W., Chen Y., Wu B., Li Z. (2021). Identification of the pyroptosisrelated prognostic gene signature and the associated regulation axis in lung adenocarcinoma. *Cell Death & Disease*.

[12] Ju A., Tang J., Chen S., Fu Y., Luo Y. (2021). Pyroptosis-related gene signatures can robustly diagnose skin cutaneous melanoma and predict the prognosis. *Frontiers Oncology*.

[13] Newman A. M., Liu C. L., Green M. R. (2015). Robust enumeration of cell subsets from tissue expression profiles. *Nature Methods*.

[14] Wang H., Luo Q., Feng X., Zhang R., Li J., Chen F. (2018). NLRP3 promotes tumor growth and metastasis in human oral squamous cell carcinoma. *BMC Cancer*.

[15] Feng X., Luo Q., Zhang H. (2017). The role of NLRP3 inflammasome in 5-fluorouracil resistance of oral squamous cell carcinoma. *Journal of Experimental & Clinical Cancer Research*.

[16] Wang H., Wang Y., Du Q. (2016). Inflammasome-independent NLRP3 is required for epithelial-mesenchymal transition in colon cancer cells. *Experimental Cell Research*.

[17] Wang Y., Kong H., Zeng X. (2016). Activation of NLRP3 inflammasome enhances the proliferation and migration of A549 lung cancer cells. *Oncology Reports*.

[18] Li L., Liu Y. (2015). Aging-related gene signature regulated by Nlrp3 predicts glioma progression. *Am J Cancer Res*.

[19] Tan Y., Chen Q., Li X. (2021). Pyroptosis: a new paradigm of cell death for fighting against cancer. *Journal of Experimental & Clinical Cancer Research*.

[20] Kovacs S. B., Miao E. A. (2017). Gasdermins: effectors of pyroptosis. *Trends in Cell Biology*.

[21] Zhang Z., Zhang Y., Xia S. (2020). Gasdermin E suppresses tumour growth by activating anti-tumour immunity. *Nature*.

[22] Jiang M., Qi L., Li L., Li Y. (2020). The caspase-3/GSDME signal pathway as a switch between apoptosis and pyroptosis in cancer. *Cell Death & Disease*.

[23] De Schutter E., Croes L., Ibrahim J. (2021). GSDME and its role in cancer: from behind the scenes to the front of the stage. *International Journal of Cancer*.

[24] Rogers C., Erkes D. A., Nardone A., Aplin A. E., Fernandes-Alnemri T., Alnemri E. S. (2019). Gasdermin pores permeabilize mitochondria to augment caspase-3 activation during apoptosis and inflammasome activation. *Nature Communications*.

[25] Erkes D. A., Cai W., Sanchez I. M. (2020). Mutant BRAF and MEK inhibitors regulate the tumor immune microenvironment via pyroptosis. *Cancer Discovery*.

[26] Rossi M. N., Pascarella A., Licursi V. (2019). NLRP2 regulates proinflammatory and antiapoptotic responses in proximal tubular epithelial cells. *Frontiers in Cell and Developmental Biology*.

[27] Courtney K. D., Corcoran R. B., Engelman J. A. (2010). The PI3K pathway as drug target in human cancer. *Journal of Clinical Oncology*.

[28] Diao M. Y., Zhu Y., Yang J. (2020). Hypothermia protects neurons against ischemia/reperfusion-induced pyroptosis via m6A-mediated activation of PTEN and the PI3K/Akt/GSK-3*β* signaling pathway. *Brain Research Bulletin*.

[29] Li L. L., Dai B., Sun Y. H., Zhang T. T. (2020). The activation of IL-17 signaling pathway promotes pyroptosis in pneumonia-induced sepsis. *Annals of Translational Medicine*.

